# Practice guidelines on migrants’ health: assessment of their quality and reporting

**DOI:** 10.1186/s12955-020-01363-7

**Published:** 2020-05-07

**Authors:** Tamara Lotfi, Mohamad I. Itani, Pamela Howeiss, Lama Kilzar, Nesrine A. Rizk, Elie A. Akl

**Affiliations:** 1 Clinical Research Institute, American University of Beirut Medical Center, P.O.Box 11-0236, Internal Medicine, Riad El-Solh, Beirut, 1107 2020 Lebanon; 2 AUB GRADE Center, Clinical Research Institute, American University of Beirut, P.O.Box 11-0236, Internal Medicine, Riad El-Solh, Beirut, 1107 2020 Lebanon; 3 Department of Internal Medicine, Faculty of Medicine, American University of Beirut, P.O.Box 11-0236, Internal Medicine, Riad El-Solh, Beirut, 1107 2020 Lebanon; 4 Faculty of Medicine, American University of Beirut, P.O.Box 11-0236, Internal Medicine, Riad El-Solh, Beirut, 1107 2020 Lebanon; 5 Department of Epidemiology & Biostatistics, Faculty of Health Sciences, American University of Beirut, P.O.Box 11-0236, Internal Medicine, Riad El-Solh, Beirut, 1107 2020 Lebanon; 6 Department of Health Research Methods, Evidence, and Impact (HE&I), McMaster University, Hamilton, ON Canada

**Keywords:** Migrants, Refugees, Clinical practice guidelines, Systematic, Quality, Health

## Abstract

**Background:**

Migrants may carry with them communicable and non communicable diseases as they move to the host country. Screening migrants may help in improving their health status and in preventing the spread of infections to the host population.

**Objective:**

To identify and assess the quality of published practice guidelines addressing migrants’ health.

**Methods:**

We included practice guidelines addressing migrants’ health at the clinical, public health or health systems levels. We searched Medline, Embase, the National Guideline Clearinghouse and the Canadian Medical Association’s Clinical Practice Guidelines Database. Two teams of two reviewers conducted in duplicate and independent manner study selection, data abstraction, assessment of the guideline quality (using the AGREE II instrument), and assessment of the quality of the reporting (using the RIGHT statement).

**Results:**

Out of 2732 citations captured by the electronic search, we included 24 eligible practice guidelines, all addressing the level of post-arrival to the host country and published between 2011 and 2017. The majority of guidelines (57%) addressed non-communicable diseases, 95% addressed screening, while 52% addressed prevention and treatment respectively. The majority of the guidelines reported their funding sources. 86% used the GRADE approach as part of the development process. The included guidelines scored high on the majority of the items, and low on the following two domains of the AGREE II instrument: rigor of development and applicability. The mean number of the RIGHT checklist items met by the included guidelines was 27, out of a total of 35. Most of the guidelines were based on systematic reviews (95.6%). A minority of the included guidelines (26%) reported considering the values and preferences of the target populations or the costs and resource implications (30%) in the formulation of recommendations.

**Conclusion:**

We identified 23 practice guidelines addressing migrants’ health, the majority of which addressed screening services. The vast majority of the captured guidelines targeted screening because the population of interest is migrants, meaning that the intention of the guidelines is to deal with additional factors than usual ones, such as prevalence of disease in country of origin, endemic diseases and others.

The guidelines suffered limitations on two quality domains (rigor of development and applicability), and have room for improvement of their reporting.

## Introduction

The United Nations define ‘migrant’ as the “person who moves to a country other.

than that of his or her usual residence for a period of at least a year [1]. There are different categories of migrants (Table 1).

**Table 1:** The five types of migrants [2]

Type of migrants	Definition
Voluntary migrants	People who have a regular visa and leave their country seeking bigger opportunities, a better job or a better education
Refugees	People who leave their country for fear of persecution for ethnic, racial or political reasons
Asylum seekers	People who seek to become refugees and waiting to be accepted in a foreign country
Asylee	A person who has been granted asylum
Parolee	A person who is temporarily accepted in a country for urgent humanitarian reasons

Patricia F Walker, M., DTM&H, FASTMHc Elizabeth D Barnett, MDWilliam Stauffer, MD, MSPH, CTropMed, FASTMH, *Healthcare for adult immigrants and refugees*, in *uptodate*. 2016

According to the United Nations, 244 million persons lived in a country other than the country where they were born in the year 2015 [3]. That represents a 41% increase compared to the year 2000 [3]. This number is expected to be as high as 405 million by 2050 [3]. In the most recent migration crisis affecting Syria, 11 million left their country since March 2011, escaping the civil war and seeking stability and refuge [4]. Worldwide, the top countries in which migrants lived in 2018 are the USA, Germany, Russia, Saudi Arabia, United Kingdom and United Arab Emirates [5].

Migration may negatively impact public health. Migrants can be exposed to abuse and exploitation affecting both their physical and mental health at various migration stages [6]. Moreover, migration can introduce new health problems to the host countries [7]. Migrants may carry to the host country both non-communicable and communicable diseases, such as tuberculosis and sexually transmitted diseases (HIV, Hepatitis B) [8].

Screening of migrants for communicable diseases can prevent their spread to the host population and reduce the apprehension of host communities regarding their introduction by migrants [7]. It can also ensure continuity of care and better management of those existing ailments [6]. On the other hand, systemic and compulsory screening may become a burden on the migrants and preclude their entry to the host country. It can negatively impact health and potentiate stigma [6]. At the same time, the financial burden on the national health system and social services will be important especially when considering the treatment of chronic diseases [6].

Practice guidelines are defined as “systematically developed evidence-based statements which assist providers, recipients and other stakeholders to make informed decisions about appropriate health interventions” [1]. Practice guidelines are important to guide clinicians and public health practitioners in addressing migrant health. The objective of this study was to identify and assess the quality of published practice guidelines addressing migrants’ health.

## Materials & methods

We systematically surveyed the medical literature for reports of practice guidelines addressing migrants’ health.

### Eligibility criteria

- Study design: guidelines meeting the WHO definition: as “systematically developed evidence-based statements which assist providers, recipients and other stakeholders to make informed decisions about appropriate health interventions” [1];
- Target population: Migrants as defined by the International Organization for Migration (IOM): “any person who is moving or has moved across an international border or within a State away from his/her habitual place of residence, regardless of (1) the person’s legal status; (2) whether the movement is voluntary or involuntary; (3) what the causes for the movement are; or (4) what the length of the stay is;” [9]
- Field: clinical practice, public health, or health policy and systems.

We did not restrict our eligibility to any specific language. When we identified two or more versions of the same guideline, we used the most recent one but kept record of all versions.

We excluded papers that did not present a set of recommendations and describe a methodology of a practice guideline. We also excluded guidelines not published in English.

### Search

We electronically searched Medline and Embase for the period of 2006–2016. We also searched both the National Guideline Clearinghouse and the Canadian Medical Association’s Clinical Practice Guidelines Database (CPGs) in November 2016.

Additional file 1 shows the full search strategies of the different databases searched. The strategies used both text words such as migrant* or emigrant* or immigrant* or refugee*, and MeSH terms such as exp. practice guidelines/, exp. migrant/, exp. refugee/;and combined terms for guidelines with terms for migrants.

### Selection process

Two teams of two reviewers screened the titles and summaries of the guidelines (when available) in duplicate and independently. Then we retrieved the full texts of the guidelines assessed as potentially eligible by at least one of the two reviewers. The two teams of two reviewers screened in duplicate and independently the full texts and compared their results. They resolved disagreements through discussion, and when needed with the help of a third reviewer. We used a standardized and pilot tested full text screening form and conducted calibration exercises at the beginning of the process.

### Data abstraction

The two teams of two reviewers abstracted data from the included guidelines, in duplicate and independently. They compared their results and resolved any disagreement through discussion and when needed with the help of a third reviewer. We used a standardized and pilot tested data abstraction form and conducted calibration exercises.

We abstracted data on the following characteristics of the guidelines:

- Year of publication;
- Type of developer organization: governmental, professional society, inter-governmental agency (e.g., World Health Organization (WHO), or other;
- Type of disease addressed: communicable, non-communicable diseases;
- Type of recommendations addressed: screening, prevention, treatment, etc.
- Type of migrants addressed (e.g., immigrants, refugees, voluntary migrant, asylum seeker, parolee);
- Setting: inpatient, outpatient;
- Migration stage addressed by: pre departure (pre-entry level), at the border (entry level), at the holding level (migrant facilities) or at the host country in the primary care [10];
- The funding source(s);
- The development methodology (e.g., use of GRADE methodology).

### Quality assessment of the guidelines

The two teams of two reviewers assessed the quality of the included guidelines using a duplicate and independent approach. They resolved their disagreements through discussion and input of a third reviewer as needed. They used the Appraisal of Guidelines for REsearch and Evaluation (AGREE) - II instrument [6]. This instrument consists of seven domains addressing different aspects of the guideline, including scope and purpose, stakeholder involvement, rigor of development, clarity of presentation, applicability, editorial independence, and overall assessment [11]. In addition, we assessed the quality of reporting using the Reporting Items for practice Guidelines in HealThcare (RIGHT) checklist [4]. The checklist includes seven sections relating to basic information, background, evidence, recommendations, review and quality assurance, funding and conflict of interests as well as other information [4].

### Data analysis

We conducted descriptive analysis for each of the variables we abstracted data for.

#### Calculation of AGREEII score

AGREE II includes six independent domains for which one quality score is calculated. Each domain includes different items, and the score per domain is the sum of the scores per item. Then this score per domain is scaled as a percentage of the maximum possible score, as shown in the formula:

$$ \mathrm{Score}\ \mathrm{per}\ \mathrm{domain}=\left(\mathrm{Obtained}\ \mathrm{score}-\mathrm{Minimum}\ \mathrm{possible}\ \mathrm{score}\right)/\left(\mathrm{Maximum}\ \mathrm{possible}\ \mathrm{score}-\mathrm{Minimum}\ \mathrm{possible}\ \mathrm{score}\right) $$

It is important to mention that high quality and low quality guidelines are not differentiated by minimum domain scores or patterns of scores across domains, but are related to user’s decisions and guided by the context of use of AGREE II [11].

#### The use of RIGHT statement

The RIGHT checklist [4] helps assessing the reporting of practice guidelines, does not recommend to calculate a score since the items are not equally weighted and scores might be problematic in research synthesis, is similar to the approach used by other reporting checklists (CONSORT and PRISMA), and is user-friendly as it states the items in the order the reader would find them when reading a guideline. Additional file 2 shows the 22 items of the checklist (with additional sub-items, making it a total of 35 combined), that assess the reporting of basic information (items 1 to 4), background (items 5 to 9), evidence (items 10 to 12), recommendations (items 13 to 15), review & quality assurance (items 16 and 17), funding and declaration and management of interests (items 18 and 19), and other information (items 20 to 22).

Our study did not require ethical approval and consent to participate since we did not collect data but used data from published papers.

## Results

**Fig. 1:**
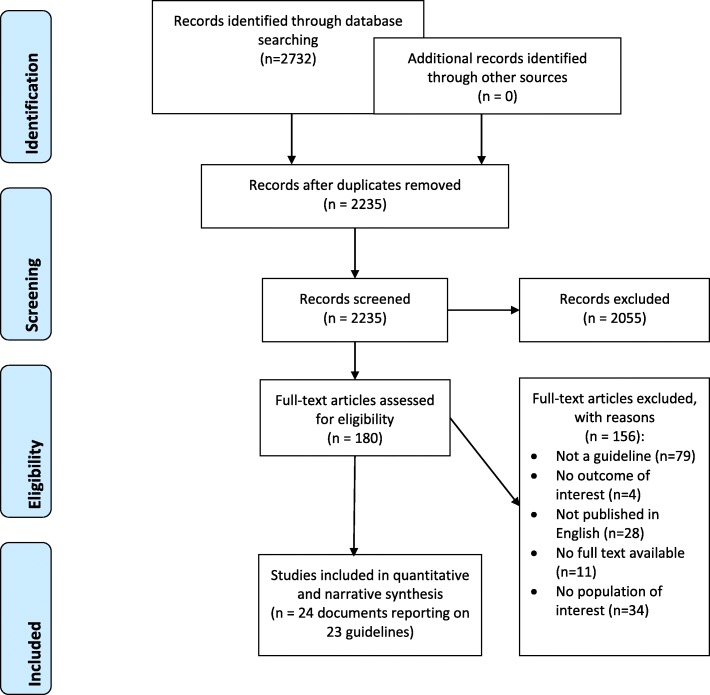
PRISMA Flow Diagram; Legend: This diagram shows the systematic process we followed to include papers captured by our search

Figure 1 shows the study PRISMA flow diagram. Out of 2732 citations captured by the electronic search, we identified 24 eligible documents reporting on 23 eligible individual guidelines. Indeed, one of the documents [12] reported additional information for 18 eligible guidelines developed under the umbrella of “Evidence-based clinical guidelines for immigrants and refugees” by Pottie et al. (2011) [12]. We excluded a total of 156 full text articles for the following reasons: 79 articles did not report guidelines, 4 were not health-related, 28 did not have full texts in English, 22 had no full texts available and 34 did not relate to migrants.

### Characteristics of guidelines

Table 2 shows the detailed characteristics of the 23 included individual guidelines.

**Table 2:** Characteristics of the included guidelines (*N* = 23^a^)

Study ID (last name of first author, year of publication)	Developer organization	Type of disease ad-dressed^b^	Type of recommendations addressed	Type of migrants addressed	Setting Migration stage addressed	Intervention	Methodology used in the development process (GRADE, …)	Funding (reported as; not reported)	AGREE II Score (%)
Kirmayer, 2011 [3]	Clinical Collaboration for Immigrant and Refugee Health (CCIRH)	NCD	• Screening using PHQ9 • Treatment	Immigrants and refugees	Outpatient: primary care	Screening using PHQ 9	GRADE	CIHR Institute of Health Services and Policy Research, the Champlain Local Health Integration Network and the Calgary Refugee Program	66.6
Dominic, 2011 (Type 2 DM)	Clinical Collaboration for Immigrant and Refugee Health (CCIRH)	NCD	• Screening using fasting blood glucose • Treatment of diabetes	Immigrants and refugees > 35 years of age from ethnic groups at high risk for diabetes type 2 (South Asian, Latin American, and African) Destination: Canada	Outpatient: primary care	Screening using fasting blood glucose And treatment	GRADE	CIHR Institute of Health Services and Policy Research, the Champlain Local Health Integration Network and the Calgary Refugee Program	71.74
Dunn, 2011 (Contraception)	Clinical Collaboration for Immigrant and Refugee Health (CCIRH)	NCD	• Screening for contraceptive needs • Counseling for contraception	Female immigrants and refugees of reproductive age Destination: Canada	Not mentioned (outpatient)	Counseling for contraception and screening	GRADE	CIHR Institute of Health Services and Policy Research, the Champlain Local Health Integration Network and the Calgary Refugee Program	67.39
Burhmann, 2011 (Vision)	Clinical Collaboration for Immigrant and Refugee Health (CCIRH)	NCD	• Screening for visual impairments • Referring to specialist	Immigrants and refugees Destination: Canada	Primary care	Screenings for visual impairment and referring to a specialist.	GRADE	CIHR Institute of Health Services and Policy Research, the Champlain Local Health Integration Network and the Calgary Refugee Program	66.66
Gagnon, 2011 (Pregnancy Screening)	Clinical Collaboration for Immigrant and Refugee Health (CCIRH)	NCD	• Screening during pregnancy	Pregnant immigrant women Destination: Canada	Not mentioned (outpatient)	Screening during pregnancy	GRADE	CIHR Institute of Health Services and Policy Research, the Champlain Local Health Integration Network and the Calgary Refugee Program	65.61
Greenaway, 2011 (Hepatitis B)	Clinical Collaboration for Immigrant and Refugee Health (CCIRH)	CD	• Screening for Hepatitis B • Treatment of Hepatitis B • vaccination against Hepatitis B	Immigrant/refugee adults and children from countries where chronic hepatitis B virus infection is moderate or high, i.e. ≥ 2% (HBsAg positive)	Not mentioned (outpatient)	Screening for Hepatitis B surface antibody	GRADE	CIHR Institute of Health Services and Policy Research, the Champlain Local Health Integration Network and the Calgary Refugee Program	63.41
Greenaway, 2011 (Hepatitis C)	Clinical Collaboration for Immigrant and Refugee Health (CCIRH)	CD	• Screening for Hepatitis C • Treatment of Hepatitis C	Immigrants coming to Canada from countries where prevalence of hepatitis C is considered high (> 3%).	Not mentioned (outpatient)	Screening for Hepatitis C Antibody And refer to a specialist if positive	GRADE	CIHR Institute of Health Services and Policy Research, the Champlain Local Health Integration Network and the Calgary Refugee Program	62.68
Greenaway, 2011 (Varicella)	Clinical Collaboration for Immigrant and Refugee Health (CCIRH)	CD	• Screening for Varicella • Vaccination against Varicella	Immigrants of all ages coming to Canada from tropical countries.	Not mentioned (outpatient)	Vaccinating against Varicella, screening	GRADE	CIHR Institute of Health Services and Policy Research, the Champlain Local Health Integration Network and the Calgary Refugee Program	66.66
Greenaway, 2011 (MMR)	Clinical Collaboration for Immigrant and Refugee Health (CCIRH)	CD	• Vaccination against MMR, Tdap and Polio	Immigrants and refugees of all ages Destination: Canada	Not mentioned (outpatient)	Vaccinating for MMR, Tdap and Polio	GRADE	CIHR Institute of Health Services and Policy Research, the Champlain Local Health Integration Network and the Calgary Refugee Program	65.58
Hassan, 2011 (Child Maltreatment)	Clinical Collaboration for Immigrant and Refugee Health (CCIRH)	NCD	• Screening for child maltreatment • Prevention child maltreatment • Treatment child maltreatment	To Canada/ From: not specified	Not mentioned (outpatient)	Screening for child maltreatment	GRADE	CIHR Institute of Health Services and Policy Research, the Champlain Local Health Integration Network and the Calgary Refugee Program	65.22
Hassan, 2011 (Intimate Partner Violence)	Clinical Collaboration for Immigrant and Refugee Health (CCIRH)	NCD	• Screening for intimate partner violence • Prevention of intimate partner violence • Treatment of intimate partner violence	Recently settled immigrant women Destination: Canada	Not mentioned (outpatient)	Preventing/ reducing morbidity and/or mortality from intimate partner violence	GRADE	CIHR Institute of Health Services and Policy Research, the Champlain Local Health Integration Network and the Calgary Refugee Program	66.67
Khan, 2011 (Parasites)	Clinical Collaboration for Immigrant and Refugee Health (CCIRH)	CD	• Screening for Strongyloides and Schistosoma • Treatment of Strongyloides and Schistosoma	Immigrants/Refugees from developing areas (Southeast Asia & Africa for Strongyloides; Africa for Schistosomiasis) Destination: Canada	Not mentioned (outpatient)	Screening and treatment for Strongyloides and Schistosoma	GRADE	CIHR Institute of Health Services and Policy Research, the Champlain Local Health Integration Network and the Calgary Refugee Program	68.11
McCarthy, 2011 (Malaria)	Clinical Collaboration for Immigrant and Refugee Health (CCIRH)	CD	• Screening for Malaria	Child and adult migrants coming to Canada from low to middle income malaria-endemic countries, especially Sub-Saharan Africa (in the past three months)	Host country Primary care	Screening for Malaria	GRADE	CIHR Institute of Health Services and Policy Research, the Champlain Local Health Integration Network and the Calgary Refugee Program	59.05
McNelly, 2011 (Dental)	Clinical Collaboration for Immigrant and Refugee Health (CCIRH)	NCD	• Screening for common oral conditions • Preventing common oral conditions • Treating common oral conditions • Referring patients to specialists	Immigrants (adults and children) coming to Canada from developed and developing countries.	Outpatient host country	Screening for dental pain and treating it with NSAIDs -Screening adults and children for dental caries and referring them to specialists	GRADE	CIHR Institute of Health Services and Policy Research, the Champlain Local Health Integration Network and the Calgary Refugee Program	a71.01
Pottie, 2011 (HIV)	Clinical Collaboration for Immigrant and Refugee Health (CCIRH)	CD	• Screening for HIV • Treating HIV	Immigrants and refugee (adolescents and adults) with countries where HIV prevalence is > 1%.	Outpatient in pre-departure countries, linkage to treatment in host countries	Screen for HIV. Linkage to treatment with counseling	GRADE	CIHR Institute of Health Services and Policy Research, the Champlain Local Health Integration Network and the Calgary Refugee Program	71.01
Pottie, 2011 (Cervical Cancer)	Clinical Collaboration for Immigrant and Refugee Health (CCIRH)	NCD	• Preventing HPV through vaccination • Screening through Cervical Cytology	Migrant women (between the age of 9 and 26 years old) coming to Canada. Source not specified.	Not mentioned	HPV vaccination. Screening for cervical abnormalities and referring for treatment.	GRADE	CIHR Institute of Health Services and Policy Research, the Champlain Local Health Integration Network and the Calgary Refugee Program	71.01
Pottie, 2011 (Iron Deficiency)	Clinical Collaboration for Immigrant and Refugee Health (CCIRH)	NCD	• Screening for Iron Deficiency • Preventing Iron Deficiency	Immigrant and refugees: 1-Children aged 1 to 4 2- female of reproductive age. -Source not reported. Destination: Canada.	Not mentioned	Screening and linkage to treatment.	GRADE	CIHR Institute of Health Services and Policy Research, the Champlain Local Health Integration Network and the Calgary Refugee Program	68.11
Rousseau, 2011 (PTSD)	Clinical Collaboration for Immigrant and Refugee Health (CCIRH)	NCD	• Screening for PTSD • Treating for PTSD	Immigrants coming to Canada	Outpatient. Host Countries	Screening for exposure to traumatic events to address functional impairment.	GRADE	CIHR Institute of Health Services and Policy Research, the Champlain Local Health Integration Network and the Calgary Refugee Program	61.23
Almasio, 2011	Italian Association for the Study of the Liver (A.I.S.F.), Italian Society of Infectious and Tropical Diseases (S.I.M.I.T.), Italian Federation Department’s Operators and AddictionServices (FederSerD), Italian Prison Medicine and Healthcare Society (S.I.M.S.Pe.).	CD	• Prevention, Diagnosis and Treatment	Migrants, Intravenous Drug Abusers and Prison Inmates	Not mentioned	Screening, Vaccinating, Treatment	Not mentioned	Not mentioned	32.61
Bhugra, 2014	European Psychiatric Association	NCD	• Screening, treatment	Migrants	Outpatient	Psychotherapy	Existing policy documents issued by relevant bodies, expert consultations, researchers and clinicians involved	Not mentioned	37.68
Yeom, 2015	Korean Society of Infectious Diseases	CD	• Prevention through vaccination against communicable diseases	Immigrants in Korea (for instance: from China, United States, Vietnam, Japan, Mongolia)	Outpatient	Vaccination	Not mentioned	Not mentioned	33.33
Chaves, 2017	The Australasian Society for Infectious Diseases and Refugee Health Network of Australia	CD & NCD	• Screening, diagnosis and management	Migrants (pediatrics and adults) asylum seeker and people from refugee like background	Not mentioned	Recommendations for testing and managing infectious and non-infectious diseases By focusing on person- centered care: asking the patient, screening him and being kind to him.	Not mentioned	The Victorian Department of Health and Human Services and ASID	65.50
Zammarchi, 2017	Coordinating resources to assess and improve HEalth status of MIgrants from Latin America (COHEMI)	CD	• Screening, diagnosis and management	Migrants and travellers	Not mentioned	Techinical Recommendations for screening, diagnosis and management of human Cysticercosis and Taenia solium taeniasis: by the COHEMI project study group	GRADE	Not reported	36.96

^a^We included the data from the Pottie et al. guideline in the guidelines it included to avoid duplicating information

^b^ CD = communicable disease, NCD = non-communicable disease

All of the included guidelines addressed the migrants in the outpatient settings, three addressed them at the host country and one at the pre departure settings. Eleven guidelines addressed strictly NCDs, ten addressed CDs and two targeted both NCDs and CDs. Almost all guidelines (95%) addressed screening, half (52%) addressed prevention, and half (52%) addressed treatment. The population described in the guidelines was “migrants” in 67% of the guidelines; “migrants, drug users and inmates” in 5%; “migrants, children and non-pregnant women of reproductive age” in 5%; “migrants and women” in 14%; “pregnant women” in 5%; and “adult migrants” in 5%. 86% of the guidelines reported using GRADE methodology to assess quality of evidence. All but three guidelines reported their funding sources [13]. The developers of these guidelines were: Canadian Collaboration for Immigrant and Refugee Health (CCIRH) (*n* = 18); joint effort between the Italian Association for the Study of the Liver (AIST), Italian Society of Infectious and tropic Diseases (SIMIT), Italian Federation Department’s Operators and Addiction Services (FederSerD), Italian Prison Medicine and Healthcare Society (SIMSPe) (*n* = 1); European Psychiatric Association (n = 1); Korean Society of Infectious Diseases (n = 1); the Australiasian Society for Infectious Diseases and Refugee Health Network of Australia (n = 1); the Coordinating resources to assess and improve Health status of Migrants from Latin America (COHEMI) (n = 1). The production of guidelines related to migrants has decreased from 19 guidelines in 2011 to two guidelines in 2017, with one guideline in 2014 and one in 2015.

### Quality of conduct

**Fig. 2:**
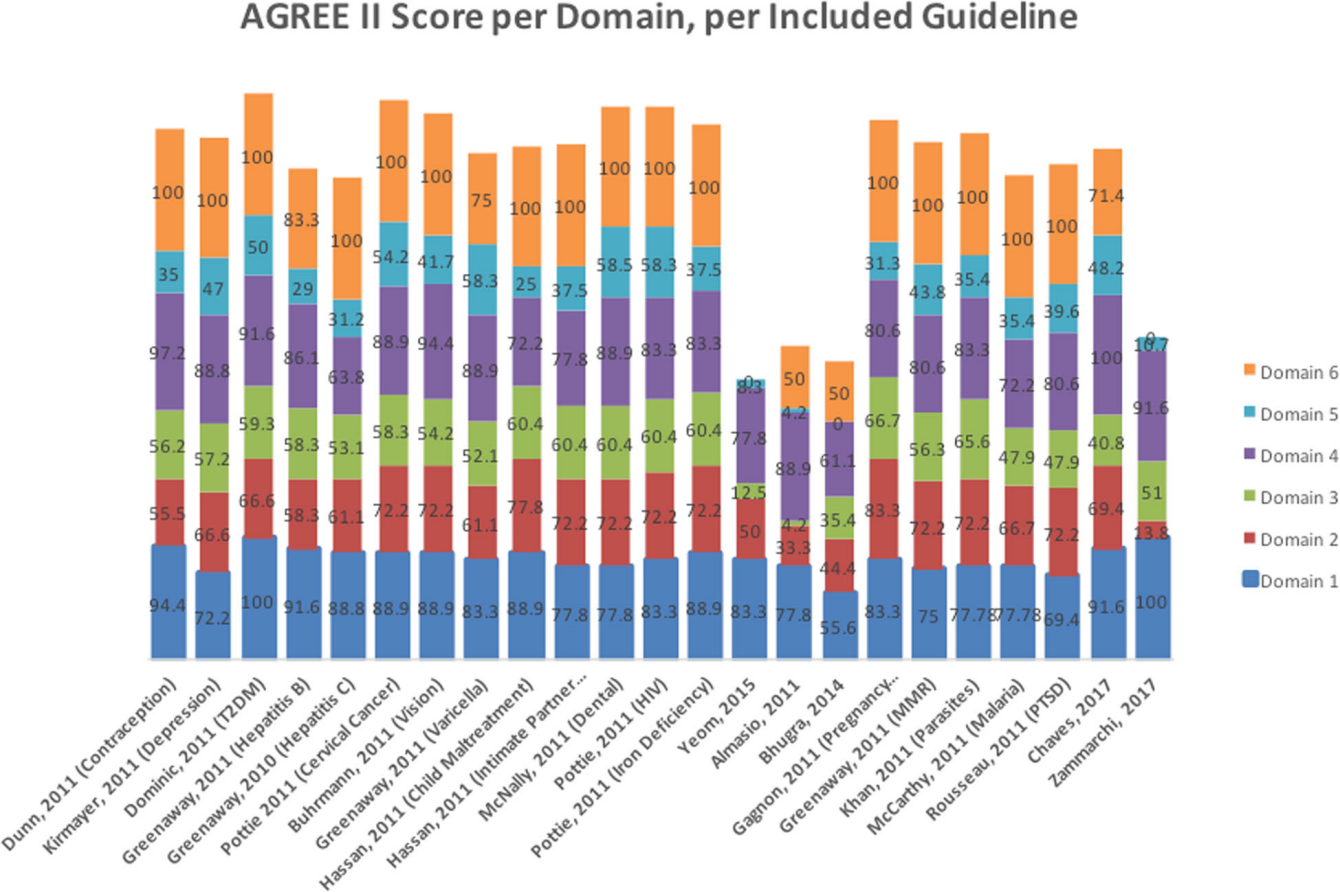
The Appraisal of Guidelines for REsearch and Evaluation (AGREE II) results per domain for each included guideline (N = 23); Legend: The X-axis: number referring to included guideline from Table B; Y-axis: percentage score on Agree II domain: Bule: Domain 1: Scope and purpose; Red: Domain 2: Stakeholder involvement; Green: Domain 3: Rigor of development; Purple: Domain 4: Clarity of presentation; Light blue: Domain 5: Applicability; Orange: Domain 6: Editorial Independence

Table 3 shows the summary score by AGREE II domains across the 23 included guidelines. Of all AGREE II domains, ‘applicability’ had the lowest median score while ‘editorial independence’ had the highest median score across the included guidelines. Most guidelines reported on conflicts of interest, and reported the role of funding bodies. Moreover, we present the scores for each of the 23 included guidelines by the AGREE II domains in Table 2 and Fig. 2. The specific reason for the low score for the rigor of development is the failure of guidelines to describe the methods used to develop the recommendations and the procedures for updates. The specific reason for the low score for applicability is the failure of guidelines to provide advice on how to apply the recommendations or monitoring criteria.

**Table 3:** Summary of the AGREE II results per domain across the included guidelines (N = 23)

Domain	Median	Q1	Q3	IQR
Scope and purpose	83.3	77.78	88.9	11.12
Involvement of stakeholders	69.4	58.3	72.2	13.9
Rigor of development	56.3	47.9	60.4	12.5
Clarity of presentation	83.3	77.8	88.9	11.1
Applicability	37.5	29.0	48.2	19.2
Editorial independence	100	75	100	25

Table 3: Summary of the AGREE II results per domain across the included guidelines (*N* = 23).

### Quality of reporting

**Fig. 3:**
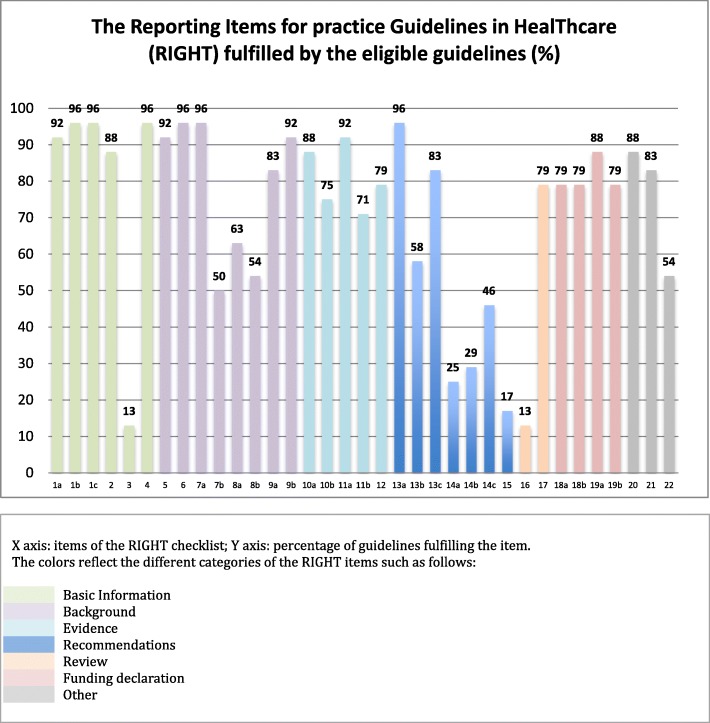
The Reporting Items for practice Guidelines in HealThcare (RIGHT) fulfilled by the eligible guidelines. Legend

Figure 3 shows the findings for all individual item of the RIGHT checklist (Appendices 2 and 3) for each of the 23 included guidelines. The mean number of items met was 27, with a minimum of 16 and a maximum of 29. The basic information (items 1–4) was properly presented in the guidelines. For the background section (items 5–9), a large majority (86.9%) of articles failed to provide proper information regarding the selection and roles of contributors (item 9a). Twelve guidelines (52%) provided separate recommendations for important subgroups (item 7b). A majority of guidelines failed to report the intended primary users of the guidelines (65%) (item 8a), or the setting for which the guideline was created (56.5%) (item 8b). The majority of included guidelines completed the items regarding the Evidence (items 10–12) overall. Most of the guidelines were based on systematic reviews (95.6%) (item 11a). As for the Recommendations (items 13–15), we judged that all of the 23 included guidelines provided clear, precise and actionable recommendations (item 13a). A minority of the included guidelines (26%) reported considering the values and preferences of the target populations (item 14a) or the costs and resource implications (item 14b) (30%) in the formulation of recommendations. Only four guidelines (17%) described the processes and approaches used by the guideline development group to make decisions (item 15). Three guidelines (13%) mentioned whether the draft guideline underwent independent review (item 16). The guidelines consistently declared funding information as well as conflicts of interests, with a section average completion of 81% (items 20–22).

## Discussion

### Summary of findings

We identified 24 documents reporting on 23 published practice guidelines addressing migrants’ health. All the guidelines were published by professional societies, between 2011 and 2017 with the majority in 2011.

The majority of the 23 guidelines (57%) addressed NCDs. Almost all included guidelines addressed screening (95%), while half tackled prevention (vaccination) (52%) or treatment (52%). Of the 23 guidelines, all mentioned the setting to be primary care (outpatient), except one that did not report the setting. 87% of the included guidelines used the GRADE methodology.

Guidelines scored high on all the quality domains of the AGREE II instrument except for two: rigor of development and applicability. The guidelines adequately met the majority of items recommended by the RIGHT checklist (74% completed at least 26/35 items).

In alignment with our findings, the World Health Organization (WHO) reported that migrants (and refugees) have similar health problems to others [14]. The most common of these problems include cardiovascular events, diabetes, hypertension, hypothermia, accidental injuries, burns, gastrointestinal illnesses and complications of pregnancy and/or delivery. WHO states that the migrants are at increased risk for NCDs due to their exposure to the “risks associated with population movements” such as drug abuse, nutrition disorders, exposure to violence, psychosocial disorders and others [14]. Moreover, the WHO states that migrants who have NCDs can be more vulnerable because of the conditions during their movements [14]..

Our findings also suggest that only half of the guidelines addressed treatment. Migrants who have lost their homes and underwent interruption of their treatment of chronic diseases will require treatment of any disease they were being treated for or have been recently diagnosed with. Guidelines can protect migrants to have their right for health ensured at hosting countries.

While one might argue that the host countries are interested in their own protection from diseases rather than caring for the health of migrants, however, one could argue that one could not expect guidelines on treatment to vary with the different populations (host and refugees). The vast majority of the captured guidelines targeted screening because the population of interest is migrants, meaning that the intention of the guidelines is to deal with additional factors than usual ones, such as prevalence of disease in country of origin, endemic diseases and others.

### Strengths and limitations

To our knowledge, this is the first study aiming to identify and assess the quality of all published practice guidelines addressing migrants’ health. This will serve researchers, healthcare practitioners, policy-makers and other stakeholders involved in caring for migrants. Moreover, we have assessed the quality of these guidelines and the quality of their reporting using standardized and validated tools. One limitation is that we did not search the grey literature (e.g., the websites of the ministries of health of migrant-hosting countries). Another is that we did not address the effectiveness of pre-screening requirements because a series of systematic reviews for different diseases was lead by the European Center for Disease Control and addressed screening measures pre and post arrival [15].

### Implications for public health practice

Refugees are considered a vulnerable population that some guidelines addressed in their recommendations, and few guidelines were developed specifically to target this population. With the increasing number of refugees and migrants worldwide, it is important to develop guidelines or adopt developed guidelines to the different contexts in order to enhance care.

Migration can directly increase the cost of appropriate management [16]. This public health burden emphasizes the necessity of developing and implementing practice guidelines that enhance the care provided to the migrants [17], or reduces the unequal quality of care for migrants compared to nationals of the hosting countries [18]. Ultimately, these guidelines will contribute to the development and enhancement of public health [19–21]. As for protection against communicable diseases, it is important for countries to have a pre-departure health assessment for potential migrants [22]. This assessment will help ensure the public health and safety of the recipient nation. In addition, it will reduce the potential burden on publicly funded health and social services [22]. Factors such as poor access to proper healthcare, low educational level, unsanitary living conditions, as well as inadequate nutrition and food hygiene interact to produce a vulnerability to infection in the migrant setting. This presents as a combination of challenges to every host country’s public health infrastructure [23]. Guidelines and public policies targeting the post-migration experiences of refugees and immigrants can minimize the negative effects of resettlement [23].

### Implications for research

First, there is a need to conduct priority setting research to identify the guidelines topics that meet the needs and expectations of both migrants and actors of the healthcare system in host countries. Second, it is important to mention that while the numbers of refugees globally are increasing, the data reported by international organizations is not always available for researchers and decision makers to use. Having local data available allows development of targeted guidelines or adaptation in order to fit the context.

Third, given that the guidelines scored low on the domain of applicability, there is a need for more research to improve the items under this domain, e.g. supporting the guideline with tools for application.

There is a need for research on the uptake and implementation of these guidelines after their publication.

## Conclusion

Our systematic survey identified 23 practice guidelines addressing migrants’ health, that focused mainly on screening. We identified methodological limitations within these guidelines particularly regarding rigor of development and applicability. We also identified a number of limitations with regards to reporting different aspects of the guidelines.

## Supplementary information

**Additional file 1.** Search Strategy.

**Additional file 2.** Checklist of the Reporting Items for practice Guidelines in HealThcare (RIGHT) items.

**Additional file 3.** Percentage of the eligible guidelines meeting each of the Reporting Items for practice Guidelines in HealThcare (RIGHT) items.

## Data Availability

Not applicable.
