# Supplementary material for: Practice guidelines on migrants’ health: assessment of their quality and reporting
Source: Health Qual Life Outcomes. 2020 May 7;18:125. doi: 10.1186/s12955-020-01363-7 (PMC7204216; doi:10.1186/s12955-020-01363-7)
Supplement: Supplementary file 1 — Additional file 1. Search Strategy. [file 12955_2020_1363_MOESM1_ESM.doc]

**Appendix 1 – Search Strategy**

Database: Embase – 1980 to October 2017

1 exp practice guidelines/

2 guideline*.ti,ab.

3 1 or 2

4 exp migrant/

5 exp refugee/

6 (migrant* or emigrant* or immigrant* or refugee*).ti,ab.

7 4 or 5 or 6

8 3 and 7

Database: Ovid MEDLINE(R) and Epub Ahead of Print, In-Process & Other Non-Indexed Citations and Daily – 1980 to October 2017

1 exp GUIDELINE/

2 guideline.pt.

3 practice guideline.pt.

4 guideline*.ti,ab.

5 1 or 2 or 3 or 4

6 (migrant* or immigrant* or emigrants).ti,ab.

7 exp "Transients and Migrants"/

8 6 or 7

9 5 and 8

Other databases:

| GIN (October 2017)  Migrant  Refugee  Immigrant |  |
| --- | --- |
| NGC (October 2017)  Migrant  Refugee  Immigrant |  |
